# Unusual phenotype of pathologically confirmed progressive supranuclear palsy with autonomic dysfunction and cerebellar ataxia: Case report

**DOI:** 10.1097/MD.0000000000005237

**Published:** 2016-11-18

**Authors:** Katerina Mensikova, Lucie Tuckova, Jiri Ehrmann, Petr Kanovsky

**Affiliations:** a Department of Neurology; b Department of Pathology, Faculty of Medicine and Dentistry, Palacky University, University Hospital, Olomouc, Czech Republic.

**Keywords:** cerebellar ataxia, multiple-system atrophy, orthostatic hypotension, progressive supranuclear palsy, tauopathies

## Abstract

**Background:**

Based on the results of recent multicenter clinical–pathological studies, it seems that the clinical heterogeneity of progressive supranuclear palsy (PSP) is much broader than previously thought. We will report 2 cases of patients with unusual manifestation of pathologically confirmed PSP.

**Methods:**

Two female patients were diagnosed with the parkinsonian phenotype of multiple system atrophy (MSAP) according to current clinical diagnostic criteria at the ages of 55 and 60 years, respectively. The patients were followed up for the next 5 and 7 years. In both cases, a detailed neuropathological examination of the brain was conducted postmortem.

**Results:**

In the first case, the overall pathological picture corresponded with the diagnosis of 4R tauopathy. In the second case, the brain pathology corresponded with a combination of 4R tauopathy and neocortical amyloidopathy.

**Conclusion:**

Some of the main symptoms of MSA, such as cerebellar ataxia and orthostatic hypotension, are not rare parts of the clinical picture of PSP. PSP can thus be mistakenly diagnosed as MSA. In order to determine the most accurate clinical diagnosis of PSP, a revision of its current clinical diagnostic criteria seems appropriate.

## 1 Introduction

Progressive supranuclear palsy (PSP) is a neurodegenerative disease of the central nervous system that may manifest with diverse clinical pictures. Its classical clinical presentation, known as Richardson syndrome (PSP-R), is characterized by progressive gait disturbance with recurrent (and frequent) falls, supranuclear vertical ophthalmoplegia, rigidity, and dysexecutive syndrome.^[1–3]^ Another phenotype, PSP-parkinsonism (PSP-P), manifests with predominant asymmetric bradykinesia, rigidity, and moderate initial response to levodopa, which may be impossible (particularly in the early stage) to distinguish from Parkinson disease.^[4]^ Another well recognized clinical variant of PSP is “pure akinesia with gait freezing”, characterized by the gradual onset of freezing of gait or speech, with the absence of rigidity, tremor, supranuclear ophthalmoplegia, or cognitive decline.^[5]^ Other recently described variants of PSP include primary nonfluent aphasia (PSP-PNFA), behavioral variant of frontotemporal dementia (PSP-bvFTD), and corticobasal syndrome (PSP-CBS),^[6–10]^ PSP-PNFA is characterized by dominant nonfluent spontaneous speech with agrammatism, phonemic paraphasia, or anomia.^[11]^ In PSP-bvFTD, behavioral and personality changes are prominent, usually meeting the diagnostic criteria for the bvFTD.^[8]^ The PSP-CBS clinical phenotype is characterized by the presence of asymmetric rigidity and apraxia, hyperreflexia, cortical sensory loss, limb dystonia, focal reflex myoclonus, alien limb phenomenon, and variable frontal lobe dysfunction.^[10]^

The definite pathological diagnosis of PSP is based on the presence of neurofibrillary tangles and neuropil threads in the midbrain structures and basal ganglia, particularly in the substantia nigra, subthalamic nucleus, and globus pallidus; a highly characteristic finding is the presence of tufted astrocytes and coiled bodies and the predominance of 4-repeat tau isoforms in the neuronal and glial tau inclusions.^[12]^ It has been hypothesized that the clinical variability of different PSP phenotypes is caused by the different distribution and density of tau pathology within the brain.^[9,10,12]^ Here, we describe 2 cases with pathologically confirmed diagnoses of PSP, in which the clinical manifestation differed from previously described clinical PSP phenotypes.

## 2 Methods

Two patients of Caucasian and Czech origin were thoroughly examined and followed up in the Tertiary Movement Disorders Center at the Department of Neurology in the Olomouc University Hospital. Clinical examinations (neurological, neuropsychological, and psychiatric) were performed, and the patients were assessed using electromyography (EMG) and evoked potentials. Magnetic resonance imaging (MRI) of the brain and spine was done in both patients. The blood and cerebrospinal fluid (CSF) samples were also examined, and the levels of inflammatory and neurodegenerative markers were assessed. Later, after the patients’ deaths, detailed neuropathological examinations of the brain were performed after the consent of the next of kin in both cases was obtained. Formalin-fixed, paraffin-embedded blocks from the following regions were obtained: frontal, temporal, parietal, and occipital cortices, hippocampus and parahippocampal region, basal ganglia, thalamus and subthalamic nucleus, midbrain at the level of the substantia nigra, pons at the level of the locus coeruleus, oblongata at the level of the inferior olive, and the cerebellum. For immunohistochemistry, 10-μm thick sections were cut from formalin-fixed, paraffin-embedded blocks. They were incubated for 1 hour with primary antibodies against the following antigens: Phospho-PHF-tau AT8 (1:200, Pierce Biotechnology, Rockford, IL, pS202/pT205), Anti-tau 3-repeat isoform RD3 (1:500, Upstate Biotechnology, Lake Placid, NY, 8E6/C11), Anti-tau 4-repeat isoform RD4 (1:300, Upstate Biotechnology, Lake Placid, NY, 1E1/A6), α-synuclein (1:5000, Signet, Dedham, MA, 4D6), β-amyloid (1:50, DAKO, Glostrup, Denmark, 6F/3D), and Anti-phospho-TDP-43 (TAR DNA-binding protein 43) (1:4000, Cosmo Bio, Tokyo, Japan, pS409/410). It was followed with Dako EnVision+ Dual Link System-HRP secondary antibody (DAKO), with incubation for 1 hour at room temperature. The immunoreactivity was visualized by liquid DAB+ substrate-chromogen system (DAKO). Finally, slides were washed under running water, dehydrated through graded ethanol, and mounted. The nuclei were counterstained with hematoxylin.

## 3 Clinical data

### 3.1 Case 1

This patient was referred for a neurological examination for the first time at the age of 55 years. She reported that the first symptoms occurred at the age of 53, when difficulties with speech and writing gradually developed; the handwriting became smaller and shaky. Neurological examination revealed dysarthria with occasional stuttering, bilateral horizontal nystagmus, bilateral pyramidal tract syndrome, and postural instability. A speech therapist characterized the speech as “extrapyramidal dysarthria”. Over the next 2 years, full parkinsonian syndrome developed, with hypomimia, asymmetric bilateral bradykinesia, hypokinesia, rigidity, bilateral resting tremor of the hands, postural instability, and repeated falls upon standing. With the further progression of the disease, the patient began to complain of difficulties with chewing and swallowing (due to the swallowing problems, there was a body weight loss of 10 kg). After 6 years of the disease course, severe dysarthria (which subsequently worsened to anarthria), bradykinesia, hypokinesia, rigidity in all 4 extremities, bilateral resting tremor of the hands, bilateral pyramidal tract syndrome, pseudobulbar syndrome, and severe postural instability with frequent falls when suddenly standing were present. There were no signs of cognitive deterioration or executive dysfunction; this fact was repeatedly confirmed by neuropsychological examination; supranuclear gaze palsy was not present.

**Figure 1:**
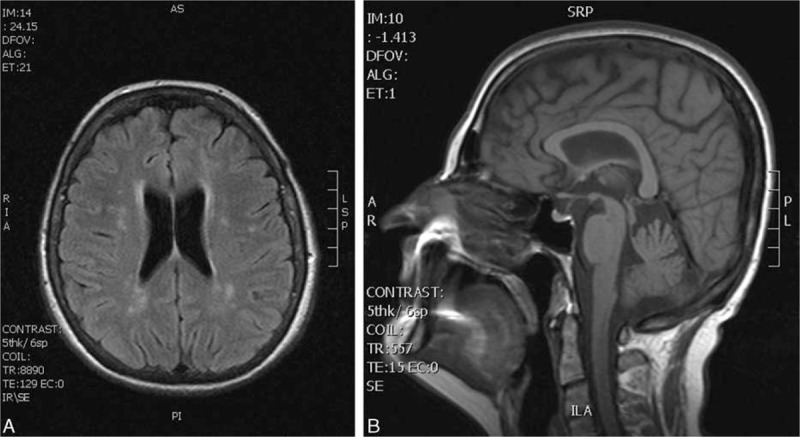
Case 1. Horizontal FLAIR sequence showing rare small nonspecific (probably postischemic) white matter lesions (A). Sagittal T1-weighted MRI, showing near-normal shape of the brainstem (B). FLAIR = fluid-attenuated inversion recovery, MRI = magnetic resonance imaging.

The bedside Thompson test revealed orthostatic hypotension, when supine blood pressure 125/90 mm Hg dropped after 3 minutes of standing to 90/60 mm Hg, which was accompanied by blurred vision and postural faintness. The head-up tilt test (80 degrees, 10 minutes) was positive; hypotension with sweating, blurred vision, and faintness developed after 4 minutes. Brain MRI (performed in the first and fourth years of the disease course) showed mild supratentorial changes in the white matter of both hemispheres, signs of the brainstem atrophy were not present (Fig. 1A and B). MRI of the cervical spine was normal. CSF examination, ultrasound examination of intracranial arteries, and neurophysiological examinations (EMG conduction studies, needle EMG, motor-evoked potentials [MEP], visual-evoked potentials [VEP], median and tibial sensory-evoked potentials [SEP], and electroencephalography [EEG]) were normal. The apomorphine test was negative. Treatment with levodopa did not improve the patient's symptoms. A diagnosis of probable multiple-system atrophy (MSA-P) was made based on the clinical diagnostic criteria (Gilman et al). A psychological examination in the seventh year of the disease course again did not show cognitive deterioration or executive dysfunction. The patient died after 7 years of illness due to cardiac arrest.

### 3.2 Case 2

**Figure 2:**
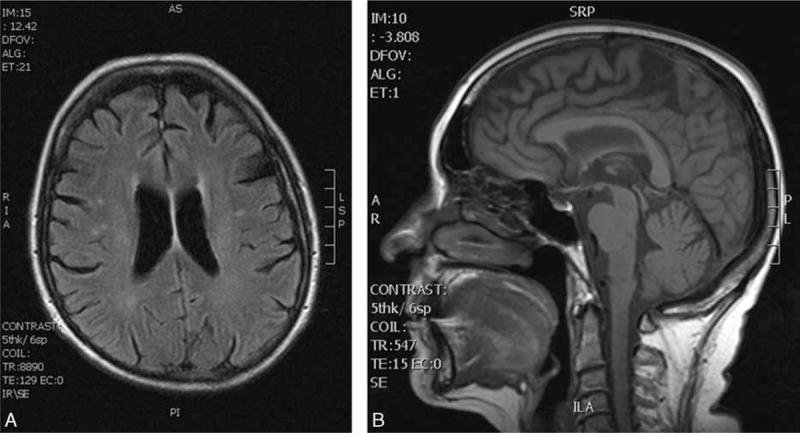
Case 2. Horizontal FLAIR sequence showing mild supratentorial changes in the white matter of both hemispheres (A). Sagittal T1-weighted MRI showing the normal shape of the brain stem (B). FLAIR = fluid-attenuated inversion recovery, MRI = magnetic resonance imaging.

This 67-year-old patient first noticed light-headedness with impaired stability when walking at the age of 60 years. These problems had lasted for 3 years when the patient was first admitted to the in-patient ward. Besides the postural instability, the patient's neurological status was normal, and ENT (Otorhinolaryngology) examination showed no significant abnormalities. MRI examination showed the brain to be normal; brainstem auditory-evoked potentials were registered with normal responses. Given the finding of moderate degenerative changes with an X-ray of the cervical spine, initial cervical myelopathy was diagnosed and the patient was referred to the department of rehabilitation medicine. Over the next 6 months, the impaired stability further progressed, and gait initiation disorder and impaired speech (dysarthria) newly appeared. Neurological examination revealed a mild ataxia with intention tremor of both upper and lower limbs, and increased elementary postural reflexes were present as a first sign of rigidity. Over the next 3 months, the dysarthria further progressed and increased urinary frequency, stiffness of the lower limbs, and repeated falls newly appeared. The patient was referred to the in-patient ward of a tertiary movement disorder center and examined there in detail. Examination showed parkinsonian syndrome with rigidity, bradykinesia and hypokinesia, shuffling gait with reduced arm swing, and positive pull-test; brisk tendon reflexes and positive Babinski sign were present bilaterally. Neuropsychological examination revealed only a mild cognitive impairment with an mini mental state examination (MMSE) score value of 28 points. MRI of the brain at that time showed only mild supratentorial changes in the white matter of both hemispheres, signs of the brainstem atrophy were not present (Fig. 2A and B). MRI of the cervical spine was normal. The bedside Thompson test revealed symptomatic orthostatic hypotension. Supine blood pressure 130/70 mm Hg dropped after 3 minutes of standing to 95/55 mm Hg, which was accompanied by dizziness and blurred vision. The head-up tilt test (80 degrees, 10 minutes) was positive; hypotension with syncope developed after 5 minutes. The urodynamic study showed apparent detrusor hyperreflexia. The biochemical, microbiological, and immunological examinations of blood serum and CSF were normal. Neurophysiological examinations (EMG conduction studies, needle EMG, MEP, VEP, median and tibial SEP, and EEG) showed no significant abnormality. The diagnosis of probable MSA-P was made based on the clinical diagnostic criteria (Gilman et al). Treatment with levodopa was initiated, but this did not improve the patient's symptoms. Over the next 12 months, the gait progressively worsened and the falls became more frequent. The patient's inability to walk and stand unaided led to the use of walkers; over the next 6 months, she became wheelchair-ridden. During the next 12 months, the rigidity and postural instability progressed, followed by the development of dystonic contractures on both upper and lower limbs bilaterally; anterocollis also appeared. Severe dysphagia, which appeared 3 months later, required a percutaneous endoscopic gastrectomy with tube feeding. As a result of the overall disease progression (mainly of the rigidity, postural instability, and dystonia), the patient gradually became immobile and bedridden; nevertheless, her cognition was still fairly good (MMSE score 27). Finally, the patient died due to bronchopneumonia after 7 years of the disease course.

## 4 Neuropathology

### 4.1 Case 1

**Figure 3:**
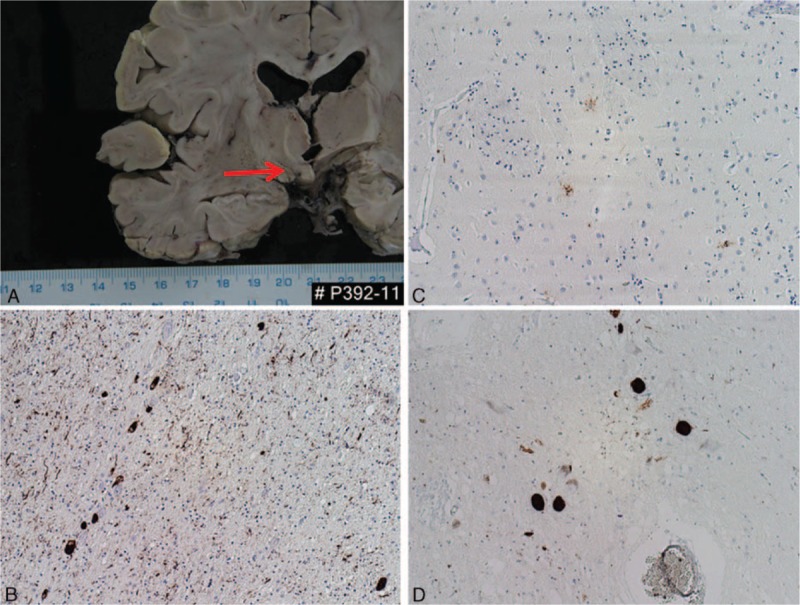
Case 1. Atrophy of the subthalamic nucleus (A). Spectrum of tau pathology in different brain regions: numerous threads, coiled bodies, and neurofibrillary tangles in the subthalamic region (AT8; B). A few tufted astrocytes in the basal ganglia (AT8; C). Neurofibrillary tangles in pigmented neurons of the substantia nigra (4RD; D). Original magnification: 100×.

The immunohistochemical examination confirmed the diagnosis of tauopathy. Mild-to-moderate positivities were seen in the reaction against phospho-tau (clone AT8) and 4R isoform of tau protein. The most prominent positivity was found in the subthalamic nucleus, which was markedly atrophic (Fig. 3A), and adjacent white matter tracts where abundant neuropil threads and coiled bodies were identified. Also, some neurofibrillary tangles, pretangles, and tufted astrocytes were seen (Fig. 3B and C). Several tufted astrocytes were well visible in the basal ganglia, together with some tangles, pretangles, and threads. A moderate-to-high degree of tau pathology was also seen in the brainstem: in the midbrain, pons, and especially oblongata (Fig. 3D). We identified numerous threads, tangles, and pretangles in the brainstem nuclei including pigmented nuclei. In the cerebellum, the tau pathology was mild to moderate. There were some threads and several neurofibrillary tangles and pretangles in the dentate nucleus. The cerebellar white matter was affected only mildly. Similarly, the hippocampus was only mildly involved. In the neocortical regions, there was a minimal pathology only in the motor cortex. The other neocortical regions were not affected at all.

In conclusion, the pathological lesions, in distribution and severity, matched the diagnosis of 4R tauopathy meeting the diagnostic criteria for PSP, scores 2 to 3 according to the Williams scoring system.^[12]^ A detailed examination excluded concomitant synucleinopathy, TDP-43 proteinopathy, or amyloidopathy.

### 4.2 Case 2

**Figure 4:**
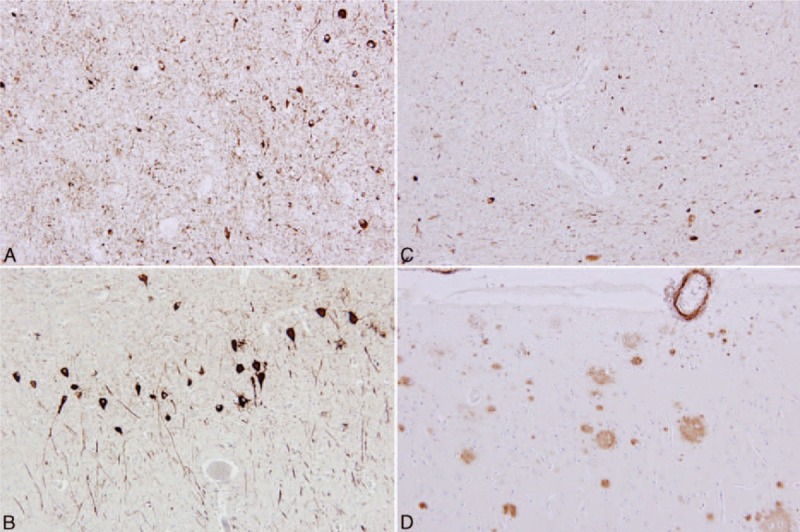
Case 2. Numerous deposits of hyperphosphorylated tau protein positive with monoclonal antibody against DR4 subtype of tau protein in the substantia nigra, proving progressive supranuclear palsy (A). Neurofibrillary degenerative structures stained with monoclonal antibody against hyperphosphorylated tau protein showing Alzheimer disease–related neuropathology (B). Numerous deposits of hyperphosphorylated tau protein positive with monoclonal antibody against DR4 subtype of tau protein in the substantia nigra, original magnification 200× (C). Different morphology of amyloid plaques and cerebral amyloid angiopathy positive in immunohistochemical reaction with monoclonal antibody against amyloid-β-peptide (D).

The immunohistochemical examination excluded a clinical diagnosis of synucleinopathy. Prominent positivities were seen in the reaction against phospho-tau protein (clone AT8) and 4R isoform of tau protein (Fig. 4A). In response to the monoclonal antibody against tau protein (clone AT8), quite numerous pretangles and tangles were evident in the hippocampus and parahippocampal region (Fig. 4B). Further, there were tufted astrocytes, neuronal cytoplasmic inclusions—neurofibrillary tangles and pretangles, and numerous threads in the brainstem especially in the substantia nigra (Fig. 4C) and pontine nuclei. The tau positive inclusions of different types were also present in the cerebellum and the basal ganglia. The motor cortex, premotor cortex, and other neocortical areas were also affected by tau pathology. Moreover, there were sparse senile plaques in the neocortex positive in the reaction with the antibody against amyloid-β peptide (Fig. 4D).

The overall pathological picture corresponded to a rare combination of 2 neurodegenerative entities: 4R tauopathy meeting the diagnostic criteria for typical PSP, scores 6 to 7 according to the Williams scoring system,^[12]^ and the neocortical stage of Alzheimer disease, reaching a low level in the ABC scoring system, A1B3C1.^[13]^

## 5 Discussion

It is currently generally accepted that the diagnosis of PSP can be definitively established only postmortem based on the characteristic histopathological findings that are in accordance with the National Institute for Neurological Disorders and Stroke—the Society for PSP criteria and on the presence of tufted astrocytes.^[14]^ The clinical diagnosis of PSP is usually made in the presence of typical symptoms such as progressive gait disturbance with recurrent falls, supranuclear vertical ophthalmoplegia, rigidity in extension, and progressive dysexecutive syndrome. As larger collections of pathologically confirmed PSP brains have been studied, it is clear that there is a range of clinical presentations of atypical PSP. Some patients with pathologically confirmed PSP have presented with CBS,^[8]^ progressive apraxia of speech,^[11]^ frontal type dementia,^[15]^ spastic paraparesis resembling primary lateral sclerosis,^[16]^ and parkinsonian features including Lewy body dementia and response to dopamine replacement therapy.^[4,17]^ This clinical variability of PSP is probably caused by the distribution of tau-pathology and its density in the different brain structures.^[9,10,12]^

The clinical picture of our first patient included a combination of parkinsonian syndrome, orthostatic hypotension with repeated falls upon standing, cerebellar ataxia with bilateral horizontal nystagmus, bilateral pyramidal tract syndrome, progressive impairment of speech leading to anarthria, and pseudobulbar syndrome. There were no signs of cognitive deficit or supranuclear gaze palsy throughout the disease course. The first symptoms developed in the patient at 53 years of age; the disease duration was 7 years. This clinical course was clearly not typical for PSP; the combination of symptoms rather met the clinical diagnostic criteria for probable MSA-P.^[18]^ The pathological changes corresponding with PSP were present in the subthalamic nucleus, basal ganglia, brainstem nuclei, oblongata, and dentate nucleus of the cerebellum. The neocortical regions were not affected, with the exception of mild pathology in the motor cortex. The presence of pathological changes in the pontine nuclei and cerebellar dentate nucleus could be related to orthostatic hypotension and cerebellar symptoms in this case.

In the second patient, the first symptoms of the disease appeared at 60 years of age. The clinical picture included progressive instability while walking, cerebellar ataxia with intention tremor of both upper and lower limbs, dysarthria, parkinsonian syndrome, autonomic dysfunction with orthostatic hypotension, increased urinary frequency, and pyramidal tract syndrome. Later, the anterocollis and dystonic contractures on both the upper and lower limbs bilaterally developed; the severe dysphagia required a percutaneous endoscopic gastrostomy with tube feeding. Only mild cognitive impairment was present after 7 years of the disease course (MMSE score 27). Apparently, the clinical picture in our second case was also not typical for PSP. The combination of symptoms fulfilled the generally accepted diagnostic criteria for probable MSA; aside from the typical combination of parkinsonism and cerebellar syndrome with autonomic dysfunction, anterocollis and contractures of the upper and lower limbs were present; these are regarded as “red flags” supporting the diagnosis of probable MSA.^[18]^

Pathological changes corresponding with the diagnosis of PSP were present in the hippocampus and parahippocampal region, substantia nigra, basal ganglia, pontine nuclei, and cerebellum and also in the motor cortex and premotor cortex; concomitant mild Alzheimer disease pathology was found along with PSP pathology. This probably did not affect the clinical manifestation, as there were no signs of significant cognitive or executive deficits, even after 7 years of the disease course. The presence of concomitant Alzheimer pathology is not rare; it has been reported in up to 36% of pathologically confirmed PSP.^[19]^ A concomitant presence of 3-repeat (RD3) isoform of tau protein is considered to be an important risk factor for developing Alzheimer pathology accompanying PSP.^[20]^ The presence of RD3 isoform of tau protein was detected in some areas of the temporal lobe in our second case. As in the first case, the presence of autonomic dysfunction and cerebellar symptoms in the clinical picture could be associated with the presence of pathological changes in the pontine nuclei and cerebellar dentate nucleus. However, the presence of cerebellar ataxia and significant orthostatic hypotension in particular led to the antemortem diagnosis of MSA in both cases.

Cerebellar ataxia is one of the main clinical symptoms of MSA, but is not considered as a typical feature of PSP. However, it may be present in cases of PSP with severe neuronal loss with gliosis and higher densities of coiled bodies in the cerebellar dentate nucleus. The clinical picture of such cases includes (besides the typical symptoms of PSP) cerebellar ataxia; it has been recently referred to as PSP-C. In a retrospective comparison of clinical symptoms of cases with pathologically confirmed diagnoses of PSP-C and MSA-C, the initial symptoms were very similar within 2 years after the disease onset and included cerebellar ataxia and falls. The principal difference between these two entities was the presence of dysautonomia and supranuclear gaze palsy, particularly in the later stages of the disease.^[21]^

Orthostatic hypotension is generally considered to be one of the cardinal symptoms of MSA, and it is also reported as one of the exclusion criteria for diagnosis of PSP.^[3]^ However, orthostatic hypotension has been described as one of the main symptoms of other neurodegenerative parkinsonian syndromes (including PSP) and was the most common reason for the misdiagnosis of MSA. In a recent work comparing clinical and pathological findings in 134 patients with in vivo diagnosis of MSA, the misdiagnosis of MSA was determined in 51 cases. Pathological diagnosis in these cases included dementia with lewy bodies (DLB), Parkinson disease (PD), and PSP. The most frequent reason for misdiagnosing DLB and PD as MSA was autonomic failure. The most frequent reason for misdiagnosing PSP as MSA was the presence of cerebellar ataxia. Several patients with PSP also had signs or symptoms of autonomic failure. This work suggests that MSA can be difficult to differentiate from DLB, PD, and PSP, not only in early stages, but also at the late stages of the disease.^[22]^ In Guadeloupean PSP (4R tauopathy) and in Guam disease with clinical characteristics resembling PSP (3R tauopathy), dysautonomia including orthostatic hypotension was present in half of the patients.^[23–25]^ Significantly high frequency of autonomic dysfunction in patients with PSP and PD was also reported in studies testing cardiovascular reflexes and other autonomic functions in patients with PSP and PD.^[26–28]^

According to the results of recent neuropathological studies of atypical parkinsonism, it is evident that mixed pathologies may be found, and cases with concomitant PSP and MSA pathology have been reported.^[19]^ In almost all of these cases, the clinical signs consistent with diagnosis of PSP dominated.^[29–32]^ In both our cases, the immunohistochemical examination excluded concomitant diagnosis of synucleinopathy.

Both our cases reflect the current view, based on the results of multicenter clinical–pathological studies, that clinical heterogeneity of PSP is much broader than it previously seemed. A substantial proportion of patients had overlapping features of atypical parkinsonian phenotypes. The combination of clinical symptoms may vary so widely that the determination of the clinical diagnosis can be difficult.^[33]^ Most phenotypes are rare in their pure form, and most patients cannot be classified into a particular phenotype, most often due to the absence of specific clinical features but also due to the presence of exclusion criteria symptoms.^[34]^ It is obvious that cerebellar ataxia and orthostatic hypotension are not rare parts of the clinical picture of PSP, so it may be difficult to differentiate from MSA, not only in early stages of the disease process, but also in the late stages. In many cases, including ours, their presence was the source of diagnostic error. In an effort to determine the most accurate clinical diagnosis of PSP, a revision of its current clinical diagnostic criteria would be appropriate.

In general, accurate clinical diagnosis in the group of atypical parkinsonian syndromes is usually difficult. It is becoming clear that current clinical diagnostic criteria are obsolete and insufficient. Symptoms stated as crucial for the individual disease entities are often lacking in their clinical picture, while symptoms which should be an exclusion diagnostic criterion may be present. In many cases, we are not able to reliably distinguish these diseases even at the level of their subcategories, that is, whether it is tauopathy, synucleinopathy, or other proteinopathy.

In our two cases actually suffering from tauopathy—PSP, was a clinical diagnosis of synucleinopathy—MSA determined. One could say that nowadays, when still there are no disease-modifying drugs available, such misdiagnosis has not any serious consequences for the patient. Currently available treatment is only symptomatic and cannot affect the course of disease itself. However, once the disease-modifying drugs are available, it will be necessary to determine the correct diagnosis in the early stage of the disease. In addition, there are other consequences: more precise differentiation at the level of individual proteinopathies could be a clue to the search for genetic mutations not only in familial but also in sporadic forms of these diseases. Another aspect would be more accurate selection of patients for enrollment into clinical trials. Molecules that have been tested in—more or less recent—trials have not proven any efficacy, and these results could be a consequence of selection of inappropriate patients for testing. Either there were selected patients suffering from diverse diseases in their early stages (in which were investigators not able to make a correct diagnosis) or there were enrolled patients with developed, well differentiated clinical phenotype in the advanced stage of the disease, when it was too late to modify the disease course.

Further research in this area should be focused on prospective multidisciplinary approach aimed to identify subtle differences in clinical phenotypes and their biological correlates. It will perhaps expand existing spectrum of clinical subtypes and their clinical presentations. Simultaneously, it will increase our ability to accurately identify and distinguish between different clinical phenotypes in their earliest stage.
